# The 3′-UTR of the adiponectin Q gene harbours susceptibility loci for atherosclerosis and its metabolic risk traits

**DOI:** 10.1186/1471-2350-14-127

**Published:** 2013-12-13

**Authors:** Nzioka Muiya, Mohammed Al-Najai, Asma I Tahir, Samar Elhawari, Daisy Gueco, Editha Andres, Nejat Mazhar, Nada Altassan, Brian F Meyer, Maie Alshahid, Nduna Dzimiri

**Affiliations:** 1 Genetics Department, King Faisal Specialist Hospital and Research Centre, P.O. Box 3354, Riyadh 11211, Saudi Arabia; 2 King Faisal Heart Institute, King Faisal Specialist Hospital and Research Centre, Riyadh 11211, Saudi Arabia

**Keywords:** Adiponectin 3′-utranslated region, Haplotypes, Metabolic syndrome, Atherosclerosis

## Abstract

**Background:**

Adiponectin Q is a hormone that modulates several metabolic processes and contributes to the suppression of biochemical pathways leading to metabolic syndrome. Hence, polymorphic changes in the adiponectin Q (*ADIPOQ)* gene are likely to contribute to metabolic disorders, and consequently lead to atherosclerosis. In the present study, we performed a population-based association study for 8 SNPs in 4646 Saudi individuals (2339 CAD cases versus angiographed 2307 controls) by real-time PCR.

**Methods:**

Linkage analysis was done by the Affymetrix Gene Chip array, sequencing by the MegaBACE DNA analysis system and genotyping accomplished by TaqMan chemistry with the Applied Biosystem real-time Prism 7900HT Sequence Detection System.

**Results:**

The rs2241766 (TG + GG) [Odds ratio(95% Confidence Interval = 1.35(1.01-1.72); p = 0.015] and rs9842733A > T [1.48(1.01-2.07); p = 0.042] were associated with hypertension [HTN; 3541 cases vs 1101 controls), following adjustment for the presence of other cardiovascular risk traits. The rs2241766 (TG + GG) was further implicated in harbouring of low high density lipoprotein levels (LHDL; 1353 versus 2156 controls) [1.35(1.10-1.67); p = 0.005], but lost its association with obesity after the adjustment for confounders. Besides, low high density lipoprotein was also linked with rs6444174 (TC + CC) [1.28(1.05-1.59)]. On the other hand, while initial univariate logistic regression analysis pointed to rs1063537 C > T (p = 0.010), rs2082940 C > T (p = 0.035) and rs1063539 G > C (p = 0.035) as being associated with myocardial infarction, significance levels of these relationships were diminished following adjustment for the influence of confounding covariates. Interestingly, haplotyping showed that an 8-mer haplotype GTGCCTCA and several of its derivatives constructed from the studied SNPs were commonly implicated in MI (χ^2^ = 4.12; p = 0.042), HTN (χ^2^ = 6.40; p = 0.011) and OBS (χ^2^ = 5.18; p = 0.023).

**Conclusion:**

These results demonstrate that the *ADIPOQ* 3′UTR harbours common susceptibility variants for metabolic risk traits and CAD, pointing to the importance of this region in atherosclerosis disease pathways.

## Background

Metabolic syndrome (MS) relates to a cluster of risk factors for atherosclerosis and type 2 diabetes mellitus (T2DM) comprising obesity, insulin resistance, hypertension (HTN) and dyslipidemia [1,2]. Adiponectin is a hormone that modulates several metabolic processes, and is believed to play a significant role in the suppression of metabolic derangements that may trigger MS. Hence, alterations in circulating adiponectin levels, largely thought to be a consequence of changes in the *ADIPOQ* gene sequence, constitute potential risk for disorders such as obesity [2], insulin resistance [2,3], T2DM [4,5], MS [3], and consequently susceptibility to acquiring atherosclerosis. In particular, two single nucleotide polymorphisms (SNPs), the rs2241766 (+45 T > G) and rs266729 (−11377C > G), among others, have been extensively discussed as important determinants of total adiponectin levels [5,6], as well as risk for T2DM [5,7], MS [3,8], obesity [8] and coronary artery disease (CAD)/myocardial infarction (MI) [9-12] in different ethnic populations. On the other hand, available literature on the role of the adiponectin gene (*ADIPOQ*) in atherosclerosis remains inconsistent. Thus, while some studies suggest a causative role for the gene [9,11-14], these findings have often turned out to be partly nonreplicable or disputable [15,16]. Hence, the essence of *ADIPOQ* as cardiovascular risk and specifically, its involvement in dyslipidaemia-related CAD onset has not been exhaustively addressed yet. Furthermore, while it is now well appreciated that early onset of CAD often manifests itself in familial dyslipidaemic disorders such as familial hypercholesterolemia (FH), the risk genes associated with this phenomenon remain to be deciphered. Specifically, the role of *ADIPOQ* in early onset CAD is still not well-understood. In a preliminary genome-wide linkage study involving a Saudi family consisting of 11 individuals, with predominant heterozygous familial hypercholesterolemia (HFH), we identified using the Affymetrix Gene Chip 250 sty1 mapping array, several genomic loci that were linked to both early onset of CAD and HFH (Additional file 1). These loci included one on chromosome 3, which harbours the *ADIPOQ* gene. Sequencing the gene in the family members and 200 individuals from the general population revealed the *ADIPOQ* gene as being extensively polymorphic in the Saudi population. This led to the notion of interactions involving some of the metabolic risk traits and the *ADIPOQ* gene as a triggering factor for both early and late onset of atherosclerosis. Furthermore, it is becoming increasingly apparent that polymorphic changes in the flanking 5 and 3 prime untranslated regions (3′UTR) of genes may exert significant influence on complex disease pathways [17,18]. In this study, we therefore set out to investigate the likelihood of some of the *ADIPOQ* variants we identified in the initial linkage experiment, conferring risk for cardiovascular and metabolic risk traits, focusing particularly on the involvement of the 3′UTR of the gene.

## Methods

### Study population

**Figure 1:**
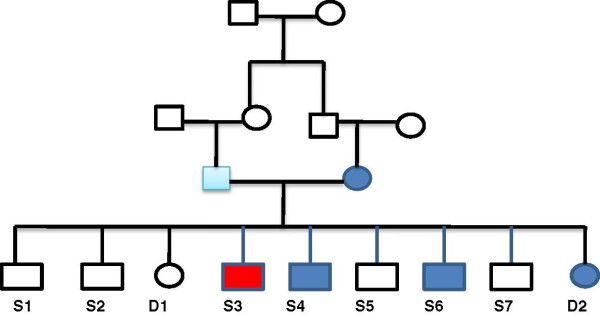
**The pedigree of the studied family.** Red denotes the primary proband (S1) and dark blue the affected members. FT, father; MT, mother; S1-7, sons 1–7; D1-2, daughters 1 and 2.

**Figure 2:**
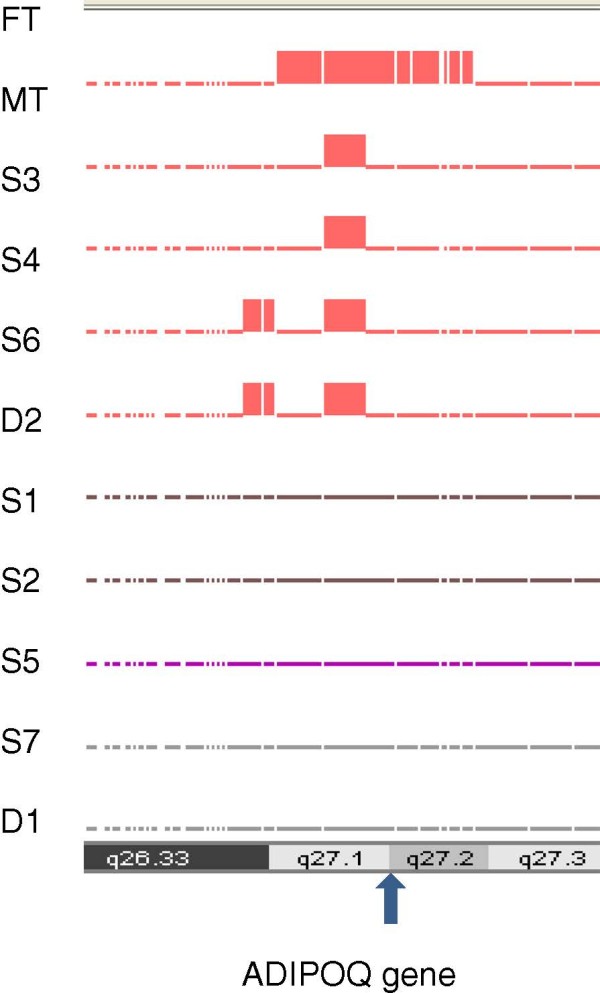
**GT console mapping indicating the position of homozygosity at the *ADIPOQ *locus for the mother (MT), and the four affected offsprings S3, S4, S6 and D2.** FT, father, MT, mother; S1-7, sons 1–7; D1 and 2, daughters 1 and 2.

The present study was performed in three parts. The initial part consisted of a linkage study on early onset of CAD in a family of 11 in which heterozygous familial hypercholesterolaemia (HFH) was rampant (Figure 1). This study linked chromosome 3q to both CAD and HFH (Figure 2). We elected to investigate the potential the role of the *ADIPOQ* gene as the most likely candidate at this locus for the development of atherosclerosis associated with the presence of metabolic risk traits in the general population. In order to identify variants of interest, we then sequenced the gene in the 11 family members and 200 individuals randomly selected from the general population. The population-based association study comprised 4646 (3045 males and 1601 females, mean age 60.3 ± 0.2 yr) angiographed individuals recruited for the population-based study on the various cardiovascular disease traits. Among these were 2339 CAD individuals (1789 males and 550 females, mean age 60.3 ± 0.2 yr) with angiographically determined narrowing of the coronary vessels by at least 50% serving as the CAD patient group (Table 1). Overall exclusion criteria were major cardiac rhythm disturbances, incapacitating or life-threatening illness, major psychiatric illness or substance abuse, history of cerebral vascular disease, neurological disorder, and administration of psychotropic medication. The control group (CON) consisted of 2307 angiographed individuals (1257 males and 1050 females, mean age 50.4 ± 0.4 yr) undergoing surgery for heart valvular diseases or those who may have reported with chest pain, but were established to have no significant coronary stenosis by angiography. Exclusion criteria for this group were, among others, ailments such as cancer, autoimmune disease, or any other disorders likely to interfere with variables under investigation. In this study population, cardiovascular risk traits for atherosclerosis, particularly HTN (n = 3541), T2DM (2559), obesity (1589) and dyslipidaemia were rampant enough to warrant independent association analyses (Table 1). Individual candidates were characterized as hypertensive if their systole/diastole blood pressure was ≥140/90 Hg [19]. Obesity was defined as having body mass index (BMI) of ≥30.0 kg/m^2^, while overweight was considered as having a BMI greater than or equal to 25 kg/m^2^ (but <30.0 kg/m^2^), according to the World Health Organization [20]. T2DM was characterized by combinations of decreased insulin secretion and sensitivity (also defined as insulin resistance) [21]. The study candidates for T2DM fulfilled the World Health Organization criteria [22] and the American Association for Diabetes Guidelines [21,23-25] for the disease. This study was performed in accordance with the regulations laid down by the Institutional Ethics Committee, and in accordance with the principles of the Declaration of Helsinki as well as Title 45, Part 46 of the U.S. Code of Federal Regulation on Protection of Human Subjects. All participants signed an informed consent.

**Table 1:** Demographics and clinical data of the studied patients

	**Controls**			**Cases**		
	**All**	**Male**	**Female**	**All**	**Male**	**Female**
**CAD**	2307	1257(54.5)	1050(45.5)	2339	1789(76.5)	550(23.5)
**MI**	1640	826(50.4)	814(49.6)	3005	2220(73.9)	785(26.1)
**FH**	3735	2451(65.6)	1284(34.4)	911	595(65.3)	316(34.7)
**T2DM**	2086	1335(64.0)	751(36.0)	2559	1710(66.8)	849(33.2)
**HTN**	1101	729(66.2)	372(33.8)	3541	2313(65.3)	1228(34.7)
**OBS**	2472	1784(72.2)	688(27.8)	1589	880(55.4)	709(44.6)
**lHDLC**	2156	1244(57.7)	912(42.3)	1762	1353(76.8)	409(23.2)
**hChol**	2019	1306(64.7)	713(35.3)	2441	1615(66.2)	826(33.8)
**hTG**	2842	1834(64.5)	1008(35.5)	1094	778(71.1)	316(28.9)
**Smokers**	2768	1310(47.3)	1458(52.7)	1759	1684(95.7)	75(4.3)
**Clinical characteristics (relative to coronary artery disease)**						
**Age**	50.4 ± 0.4	51.2 ± 0.5	49.8 ± 0.5	60.3 ±0.2	59.8 ± 0.3	61.8 ± 0.54
**BMI**	29.0 ±0.2	27.97 ± 0.2	30.3 ± 0.3	28.9 ± 0.1	28.3 ± 0.1	31.0 ± 0.3
**Total Chol**	4.51 ± 0.02	4.42 ± 0.03	4.62 ± 0.03	4.48 ± 0.02	4.43 ±0.03	4.66 ± 0.05
**HDL-Chol**	1.26 ±0.01	1.18 ±0.03	1.33 ±0.01	1.15 ±0.01	1.15 ± 0.01	1.25 ± 0.02
**LDL-Chol**	2.76 ± 0.02	2.73 ± 0.03	2.80 ±0.03	2.71 ±0.02	2.68 ± 0.02	2.84 ±0.06
**TG**	1.52 ± 0.02	1.60 ± 0.03	1.44 ± 0.03	1.78 ± 0.02	1.78 ± 0.03	1.78 ± 0.05
**FG**	6.87 ± 0.16	6.80 ± 0.23	6.92 ± 0.22	9.45 ± 0.31	9.27 ± 0.37	9.88 ± 0.56
**BP**	120/83	119/81	121/82	128/84	130/85	127/83

The numbers in brackets give the fraction of the total (all) values of the group. BMI, body mass index; BP, blood pressure; CAD, coronary artery disease; Chol, cholesterol; FG, fasting glucose; FH, family history of CAD; MI, myocardial infarction; TG, triglycerides; T2DM, type 2 diabetes mellitus; hLDLC, high low density lipoprotein-cholesterol; hChol, hypercholesterolaemia; HDL, high density lipoprotein; hTG, hypertriglyceridaemia; HTN, hypertension; LDL, low density lipoprotein; lHDLC, low high density lipoprotein-cholesterol; OBS, obesity.

#### Linkage analysis for early onset coronary artery disease

Five ml of peripheral blood was sampled in EDTA tubes from each of the study individuals after obtaining their written consent, and genomic DNA extracted from leukocytes by the standard salt methods using PUREGENE DNA isolation kit (Gentra system, Minneapolis, MN, USA). Genome-wide scanning was performed using the Affymetrix Gene Chip 250 sty1 mapping array (Affymetrix, Inc., Santa Clara, CA, USA). Briefly, 250 ng of genomic DNA was digested with the restriction endonuclease StyI, mixed with its adaptors and ligated with T4 DNA ligase. The mixture was added to four separate PCR reactions, amplified, pooled and purified to remove the unincorporated dNTPs. The PCR product was fragmented, biotinylated, hybridized to the 250 sty1 array for 18 h, washed, stained and scanned as recommended by the manufacturer. SNP genotypes, linear chromosomal locations and marker ordering were detected with the Affymetrix GeneChip® Genotyping Analysis Software (GTYPE) Version 4.0.

Multipoint parametric linkage analysis was performed using the GeneHunter Easy Linkage analysis software 4.0 (Affymetrix, Inc., Santa Clara, CA, USA) for estimating the LOD scores. Both autosomal recessive and dominant models of inheritance were tested in search of chromosomal linkage regions, using Asian SNP allele frequencies. Copy Number Analyzer for GeneChip® (CNAG) Ver. 3.0 (Affymetrix, Inc., Santa Clara, CA, USA) was employed to check the shared chromosomal regions for homozygosity.

#### Screening for ADIPOQ mutations

The *ADIPOQ* gene was sequenced using the MegaBACE DNA analysis system (Amersham Biosciences, Sunnyvale, CA, USA). Briefly, the DNA was subjected to PCR by standard methods described elsewhere. Five μl of PCR product were treated with 2 μl of ExoSAP-IT (USB Corporation, Cleveland, Ohio, USA) at 37°C for 30 min to allow the hydrolytic removal of excess primers and dNTPs by Exonuclease 1 and Shrimp Alkaline Phosphatase. The enzymes were inactivated at 80°C for 15 min, and the sequencing reaction was initiated by mixing 2 μl DNA, 1 μl of 5 μmol primer, 8 μl of DYEnmic ET Dye Terminator (Amersham Biosciences, Buckinghamshire, UK) and 9 μl of distilled water. The mixture was thermally cycled 40x at 95°C for 20 sec, 50°C for 15 sec, and 60°C for 1 min. Unincorporated dye-labelled terminators were removed by gel-filtration through the DyeEx 96 plate (Qiagen GmbH, Hilden, Germany). The eluent was vacuum-dried and dissolved in 10 μl of loading solution (GE Healthcare UK Ltd, Little Chalfont, Buckinghamshire UK) for sequencing. Data were analyzed for SNPs by Lasergene software (DNASTAR, Inc. Madison, WI, USA).

#### Association studies

Five ml of peripheral blood was sampled in EDTA tubes from each of the study individuals after obtaining their written consent, and genomic DNA extracted from leukocytes by the standard salt methods using PUREGENE DNA isolation kit (Gentra system, Minneapolis, MN, USA). Genotyping was achieved using the Taqman chemistry with the ABI Prism 7900HT Sequence Detection System (ABI Inc., Foster City, CA, USA). Primers and the TaqMan fluorogenic probes bearing a suitable reporter dye on the 5′-end and a quencher dye on the 3′-end were designed using the Primer Express software V2.0 (ABI Inc., Foster City, CA, USA) and procured from Applied Biosystems (ABI, Warrington, UK). One probe was labeled with the VIC dye and the other with the FAM dye at the 5′-end, and serial dilutions were run to determine the optimal working concentrations. For each reaction, a 25 μl reaction mixture was prepared from 5 μl containing 50 ng DNA, 12.5 μl of 2x Universal mix (Eurogentec, Liege Science Park, Seraing, Belgium), 1.25 μl of 20x probe assay mix and 6.25 μl DNase-free distilled water. Three no-template controls were included in each plate for normalization of the emission signal. The first two cycles occurred at 50°C for 2 minutes, and 95°C for 10 min, followed by 40 amplification cycles of 94°C for 15 sec, and 60°C for 30 sec. The plates were then scanned for FRET signal using the 7900HT Sequence Detection system and data analyzed with SDS 2.4 software (ABI, Foster City, CA, USA).

#### Statistical analysis

The power calculation for the statistical validity of our study population was calculated using the following standard formula, $n=\frac{Z^{2}p\left(1-p\right)}{d^{2}}$ whereby ***Z*** is generally set to 1.96, ***p*** is the conversion rate expected to be observed (e.g. 0.7), and ***d*** represents the minimum absolute size difference between the two groups. Using this formula, we estimated that the sample size (***n***) required for our study would be 323. In the present study, we employed a population of 4646, which was large enough to produce statistically meaningful interpretation of our results. Comparison of genotypes and alleles between different groups for continuous dependent variables was achieved by Analysis of Variance (ANOVA) or Student’s test as appropriate. Categorical variables were analyzed by Chi-Square test. Univariate and multivariate logistic regression analyses were used to compute odds ratios and their 95% confidence intervals, using the SPSS software version 20 (SPSS Inc., Chicago, USA). The haplo.stats package (http://mayoresearch.mayo.edu/mayo/research/schaid_lab/software.cfm) in the R Statistical Computing software (http://www.r-project.org/) was used to perform haplotype-based association analysis. Significance of association was determined between haplotypes and the case–control status - a binomial trait denoting whether or not a patient carried the disease. Odd ratios were calculated using as reference the baseline haplotype AGGAGAGA, and the Haplotype Score statistics for the association of a haplotype with the binary traits calculated as in Schaid *et al.*[26] and Lake *et al*. [27], whereby an association with a two-tailed *p* value <0.05 was considered statistically significant.

## Results

### Linkage analysis and gene sequencing

The sequencing of all exons, exon-intron junctions, the promoter as well as the 3′-UTR of the *ADIPOQ* gene in the HFH family members and 200 other individuals from the general population led to the discovery of various single nucleotide changes. The data was interrogated against the HapMap (http://hapmap.org) and Perlegen (http://genome.perlegen.com) databases to identify single nucleotide polymorphisms (SNPs) of potential interest. A total of 8 SNPs [rs2241766 (1), rs6444174 (2), rs6773957 (3), rs1063537 (4), rs2082940 (5), rs4686804 (6), rs1063539 (7) and rs9842733 (8)] were then selected for further evaluation in the general population involving 4646 individuals. All of these SNPs reside in the 3′UTR, except the rs2241766, found in exon 1 of the gene. This SNP was included based on the inconsistency in current literature discussing its potential impact as a cardiovascular disease risk as well as the high prevalence of its heterozygosity in our HFH study family.

### Association studies

#### Adiponectin Q genotyping and cardiovascular risk traits

We first evaluated the relationship of the *ADIPOQ* variants with CAD/MI and its risk traits in our general population. The clinical demographics of the studied patients are given in Table 1. Two of the studies variants, the rs2241766 (TG + GG) [Odds ratio(95% Confidence Interval = 1.35(1.01-1.72); p = 0.015] and rs9842733A > T [1.48(1.01-2.07); p = 0.042] were associated with hypertension (HTN; 3541 cases vs 1101 controls), following adjustment for the presence of other risk factors. The rs2241766 (TG + GG) was further implicated in harbouring of low high density lipoprotein levels (LHDL; 1762 versus 2156 controls) [1.35(1.10-1.67); p = 0.005), but lost its association with obesity after the adjustment. Besides, LHDL was also linked with rs6444174 (TC + CC) [1.28(1.05-1.59). However, while univariate logistic regression analysis pointed to rs1063537 C > T (p = 0.010), rs2082940 C > T (p = 0.035) and rs1063539 G > C (p = 0.035) as being associated with myocardial infarction, the significance of these relationships was lost following adjustment for the influence of other covariates (Table 2 and Additional file 1).

**Table 2:** Adiponectin Q genotyping in cardiovascular disease traits in the general population

**Variant**	**Controls**	**Cases**	**Uncorr. B.(95% CI)**	**Uncorr. P-value**	**Corr. B.(95% C.I.)**	**Corr. P-value**
**Hypertension**						
rs2241766 (TG + GG)	0.844	0.855	1.25(1.01–1.56)	0.039*	1.35(1.01–1.72)	0.015*
rs9842733 T	0.019	0.028	1.44(1.03–2.01)	0.031*	1.48(1.01–2.07)	0.042*
**Myocardial Infarction**						
rs1063537 T	0.165	0.186	1.16(1.04–1.30)	0.010**	1.16(1.04–1.30)	0.070
rs2082940 T	0.206	0.225	1.12(1.01–1.24)	0.035*	1.11(0.98–1.26)	0.102
rs1063539 C	0.176	0.194	1.13(1.01–1.20)	0.035*	1.13(1.01–1.20	0.091
**Hypercholestrolaemia**						
rs9842733 T	0.023	0.030	1.32(1.01–1.72)	0.043*	1.38(0.97–1.69)	0.085
**Low HDL-Cholesterol**						
rs6444174 (TC + CC)	0.848	0.855	1.23(1.08–1.49)	0.042*	1.28(1.05–1.59)	0.016*
rs2241766 (TG + GG)	0.847	0.858	1.32(1.06–1.59)	0.013*	1.35(1.10–1.67)	0.005***
**Obesity**						
rs2241766 (TG + GG)	0.848	0.858	1.27(0.96–1.54)	0.028*	1.61(0.99–1.54)	0.67

The table lists the variants associated with the hypertension (3541 cases vs 1101 controls), myocardial infarction (3005 vs 1640), obesity (1589 vs 2472), hypercholesterolaemia (2441 vs 2019) and low high density lipoprotein (1762 vs 2156) among the 4646 studied individuals before and following adjustments for the influences of other risks factors. Bonferroni corrections for age, sex and family history was performed for all studied variables, while inclusion of the individual disease traits into the multivariate analysis varied dependent on the primary study case group. B, Coefficient; C.I., confidence interval; Uncorr. P-value; uncorrected P-value; Corr. P-value; the adjusted P-values for the associations in presence of other factors. *P < 0.05, **P < 0.01, ***P < 0.005 by χ^2^ test.

#### Adiponectin Q haplotyping and disease

**Figure 3:**
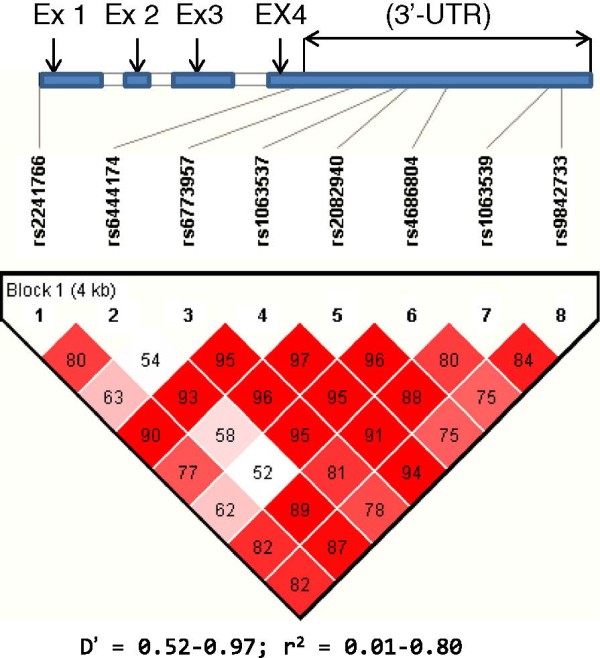
**Linkage disequilibrium structure of the eight studied single nucleotide polymorphisms.** Ex, exon; 3′-UTR, 3 prime untranslated region; *ADIPOQ*, adiponectin Q gene; D’ = linkage disequilibrium coefficient; r = regression coefficient.

Having established the sharing of a number of the *ADIPOQ* variants by different disease traits, the next obvious stage was to ascertain whether or not similar relationships for the studied SNPs could be manifest at haplotype level. Thus, we employed all the 8 studied SNPs to construct haplotypes of potential interest (Figure 3). Accordingly, using the most common 8-mer haplotype TTACCCCA (frequency = 0.344) as baseline for the comparative analysis, we found several haplotypes that were significantly associated with the various disease traits, as listed in the *ADIPOQ* (Haplo Suppl data, Additional file 2: Table S4, and summarized in Table 3). Notably, by far the highest number of haplotypes was linked to MI. These included the 8-mer GTGCCTCA (χ^2^ = 4.13; p = 0.042) and several of its derivatives, such as the 7-mer GTGCCTC (block 1–7; χ^2^ = 4.41; p = 0.036), the 6-mer GTGCCT (block 1–6; χ^2^ = 4.40; p = 0.036), the 5-mer GTGCC (block 1–5; χ^2^ = 4.86; p = 0.028) as well as the 4-mer GTGC (block 1–4; χ^2^ = 5.06; p = 0.025) which were equally involved in a causative fashion. In-depth analysis indicated that the 4-mer haplotype GTGC, constructed from rs2241766, rs6444174, rs6773957 and rs106353, was the core for this relationship. Interestingly, apart from MI, the same 8-mer haplotype GTGCCTCA also conferred risk for HTN (χ^2^ = 6.40; p = 0.011) and obesity (χ^2^ =5.18; p = 0.023), revealing a common causative genomic sequence for these three important cardiovascular risks traits at the haplotype level (Additional file 2: Table S6). Moreover, for the HTN subset, it was the 7-mer GTGCCTC (block 1–7; χ^2^ = 7.17; p = 0.007) and 6-mer GTGCCT (block 1–6; χ^2^ = 7.188; p = 0.007), constructed from the SNPs in the 3′UTR, which exhibited the most significant relationship. Thus, this observation unequivocally demonstrates that the observed changes in the 3′UTR are indeed crucial, not only at single nucleotide, but also genomic sequence levels, in events leading to these disease traits.

**Table 3:** Sharing of adiponectin Q haplotypes by cardiovascular risk traits

**SNP block**	**Haplotype**	**Trait**	**Pooled**	**Controls**	**Cases**	**χ**^**2**^	**P-value**
1-8	GTGCCTCA	MI	0.025	0.022	0.029	4.13	0.042
		HTN	0.025	0.021	0.029	6.40	0.011**
		OBS	0.024	0.020	0.027	5.18	0.023*
1-7	GTGCCTC	CAD	0.025	0.021	0.030	7.17	0.007**
		MI	0.025	0.023	0.030	4.41	0.036
		HTN	0.025	0.021	0.030	7.17	0.007**
		OBS	0.025	0.020	0.028	6.19	0.013**
1-6	GTGCCT	MI	0.025	0.023	0.030	4.40	0.036
		HTN	0.025	0.021	0.030	7.19	0.007**
		OBS	0.025	0.020	0.028	5.51	0.019**
1-5	GTGCC	MI	0.027	0.025	0.032	4.86	0.028
		HTN	0.027	0.023	0.032	6.46	0.011**
		OBS	0.026	0.022	0.029	4.22	0.040
1-4	GTGC	MI	0.028	0.025	0.033	5.06	0.025
		HTN	0.028	0.023	0.032	6.60	0.010**
		OBS	0.027	0.023	0.030	4.19	0.041
5-8	TCCA	OBS	0.025	0.029	0.022	4.95	0.026
		lHDL	0.025	0.021	0.029	5.06	0.025

The table lists some of the shared haplotypes constructed from the studied variants, rs224176 (1), rs6444174 (2), rs6773957 (3), rs1063537 (4), rs2082940 (5), rs4686804 (6), rs1063539 (7) and rs9842733 (8), arranged sequentially by their chromosomal positions. The most frequent 8-mer haplotype TTACCCCA (0.344) was employed as the baseline to determine the relative effects of these haplotypes. The pooled, control and case haplotype frequencies are proportions. SNP blocks represent the range of variants constituting the respective haplotypes. CAD, coronary artery disease; MI, myocardial infarction; HTN, hypertension; OBS, obesity; lHDL, low high density lipoprotein. *P < 0.02 and **P < 0.01 by χ^2^ test.

Interestingly, in contrast to the above traits, dyslipidaemic disorders were linked only to shorter haplotypes, which were for the most part different from those implicated in the other diseases. Thus, elevated cholesterol levels were significantly associated with two sequences, the 5-mer TTATT (χ^2^ = 9.56; p = 0.002) and its 4-mer derivative TTAT (χ^2^ = 7.56; p = 0.006) involving the SNPS rs2241766, rs6444174, rs6773957 and rs10635, while LHDL levels were only linked to the 4-mer TCCA (χ^2^ = 5.06; p = 0.025) (Table 3). It should also be mentioned that several protective haplotypes were partly shared by the disease traits, including the 8-mer GTGCCTCA and its derivatives, which were protective against both HTN (χ^2^ = 6.40; p = 0.011) and obesity (χ^2^ = 5.18; p = 0.023). Further analysis also indicated that the core for these protective properties was the complementary nucleotides of the same risk SNPs.

## Discussion

The present study was designed to initially evaluate possible genomic linkage to early onset of CAD in HFH, which resulted in the identification of several genomic loci implicated in both disorders. These loci included one on chromosome 3, which harbours the *ADIPOQ* gene, an attractive candidate for predisposing individuals to cardiovascular disease. Ensuing sequencing of the gene in the study family and general population revealed several informative SNPs of potential interest, including some residing in the 3′UTR of the gene. Hence, we elected to focus primarily on the essence of genomic changes in this region as a contributing factor to disease pathways leading to the manifestation of atherosclerosis.

To begin with, three of the studied SNPs, rs1063537, rs2082940 and rs1063539, appeared to be associated with CAD/MI in the univariate analysis, albeit turning non-significant in the presence of confounders, such as age, gender, family history and other disease traits. However, at least two variants were associated with the degree of atherosclerosis, indicating indeed that changes in the *AQIPOQ* gene sequence pose a calculable risk for the disease processes involved in the development of atherosclerosis. To our knowledge, hardly any data exists yet in the literature on the influence of these SNPs on CAD/MI disease pathways, pointing to the novelty of our present findings. Besides, although our study did not identify any delineable relationship for four other studied variants with CAD/MI per se, several other investigations including some genome-wide association studies have implicated these SNPs as well as the *AQIPOQ* locus in general, in the disease [12,14,28]. Thus, our results furnish support to the notion of the *ADIPOQ* as a risk gene for atherosclerotic processes.

Perhaps one of the most well-studied among these SNPs is the rs2241766 (+45 T > G), which has been associated with CAD/MI in different ethnic groups, including the Iranian and Chinese Han populations [11]. Interestingly, our study also established significant correlation for this SNP with HTN and the harbouring of LHDL-cholesterol levels, adding weight to the notion of common loci in this genomic region as an underlying cause for these atherosclerosis risk traits. The fact that this SNP was associated not only with these two diseases, but also weakly implicated in obesity, strengthens the notion of its candidacy as a risk factor for these important cardiovascular risk traits in our population. In fact, its relationship with MI turned significant within these disease subsets, except obesity, suggesting a contributory component of these traits on the influence of this genic change on atherosclerotic disease pathways. Notably, this variant has also been associated with elevated serum adiponectin levels in the Finnish and Thai populations [5,29]. However, the influence of the rs2241766 on these different disease traits or changes in adiponectin levels could not be replicated in a number of individual studies as well as meta-analyses [15,16]. This scenario points to potential ethnic differences in the way this variant influences these disease traits. Indeed, gender, ethnicity and classical confounders have been suggested as accounting for some differences in the observed relationships of some of the various *ADIPOQ* variants with disease or elevation in its protein serum levels. Thus, for example, the rs2241766 has been implicated in higher body mass index (BMI) in absence of alterations in blood lipids, blood pressure or obesity in Iranian females, leading to the notion of gender-selective effect of the genotypes on BMI [30]. It is also worth mentioning that the rs2241766 constitutes a silent polymorphism, which would suggest that no change in protein function is linked to it. However, it has been suggested recently that many organs possess the ability to selectively use certain codons for translational stability, transcriptional selection, RNA stability, or protein hydropathy, for example [31,32]. Hence, the finding of an association of this SNP with disease in this and other studies seems to point to the essence of this genomic locus with respect to such regulatory mechanisms.

Amazingly, the sharing of causative entities was manifest not only at SNP, but also at haplotype level, whereby several 8-mers constructed from all the studied SNPs as well as their derivatives conferred risk for the individual disease traits. Moreover, the significance levels for some of the haplotype relationships were much greater than those for the individual SNPs, pointing to the strength of haplotyping versus genotyping as a more robust indicator for disease. Hence, our present observations raise the all-important question as to whether or not there exists some form of *ADIPOQ* gene-disease trait interactions paving the pathway(s) to atherosclerosis that are related to these manifestations. Interestingly, in addition to predicting the extent of the disease, the rs9842733 T was also implicated in HTN and hChol, linking severity of atherosclerosis with these important metabolic risk traits at the level of single nucleotide alterations, intuitively providing further support for such a probability. These findings therefore indicate that *ADIPOQ* polymorphism may offer some genetic explanation for common disease pathways associated with atherosclerosis.

Mechanistically, an important aspect of the present study is that 7 of the 8 investigated SNPs reside in the 3′UTR of the gene, and are invariably implicated in various diseases traits predisposing individuals to atherosclerosis. Notably, exclusion of the rs2241766 from the haplotype constructs did not alter the impact of the 7-mer derivatives constructed from the 3′UTR variants, pointing to a pivotal role for this genic region in our observations. These findings demonstrate the importance of this region in pathways linking atherosclerosis with its disease risk traits. In this regard, it is increasingly acknowledged that the 3′UTR region contains important elements, such as micro RNAs, that are involved in gene regulatory processes, such as mRNA maturation. While our results do not warrant qualified speculation as to what mechanisms may be involved, it can nonetheless be concluded that defects in such processes may be important for gene-trait interactions leading to complex disorders, such as atherosclerosis. In fact, the candidacy of the 3′UTR as a genomic risk locus for atherosclerosis remains to be exploited, and therefore our notion calls for further studies of this subject.

## Conclusions

Our study described the sharing of several *ADIPOQ* variants by metabolic risk traits among themselves as well as CAD/MI, in support of an important role for this gene in atherosclerosis disease pathways. Most important, the results also point to the harbouring by the *ADIPOQ* 3′UTR of important elements regulating common atherosclerosis disease pathways.

## Abbreviations

ADIPOQ: Adiponectin Q gene; BMI: Body mass index; CAD: Coronary artery disease; FH: Family history; hChol: Hypercholesterolaemia; hTG: Hypertriglyceridaemia; HTN: Primary hypertension; OBS: Obesity.

## Competing interest

The authors declare that they have no competing interests.

## Authors’ contributions

NM was involved in running the Affymerix assays, designing probes, screening for gene mutations as well as participating in the write up of the manuscript, SMW was responsible for mapping studies, MN was responsible for overall running the TaqMan assays, AIT performed part of the statistical analysis, SE performed part of the sequencing experiments, DG ran part of the TaqMan assays, EA was responsible for clinical patient data and material acquisition, NM was responsible for patient recruitment and sample collection, MA supervised the recruitment of the patients and compliance with Institutional ethical procedures, NA assisted in performing the statistical analysis, BFM contributed to the designing of the experiments and write up of the manuscript; ND is the Principal Investigator, with the overall responsibility for the project and preparation of the manuscript. All authors read and approved the final manuscript.

## Pre-publication history

The pre-publication history for this paper can be accessed here:

http://www.biomedcentral.com/1471-2350/14/127/prepub

## Supplementary Material

Additional file 1 ***ADIPOQ *** **Suppl data.** Click here for file

Additional file 2 ***ADIPOQ *** **Haplo Suppl data.** Click here for file
