# Supplementary material for: The 3′-UTR of the adiponectin Q gene harbours susceptibility loci for atherosclerosis and its metabolic risk traits
Source: BMC Med Genet. 2013 Dec 13;14:127. doi: 10.1186/1471-2350-14-127 (PMC3925068; doi:10.1186/1471-2350-14-127)
Supplement: Additional file 2 — ADIPOQ Haplo Suppl data. [file 1471-2350-14-127-S2.docx]

**Supplementary Tables**

| **Suppl. Table 1:** Important clinical characteristics and genotyping of the studied heterozygous familial hypercholesterolaemia family | | | | | | | | | | | | | | | |
| --- | --- | --- | --- | --- | --- | --- | --- | --- | --- | --- | --- | --- | --- | --- | --- |
| **Relationship** | **Sex** | **Age** | **Status** | **Chol** | **Triglycerides** | **LDL-Chol** | **HDL-Chol** | **rs 2241766_TG** | **rs 6444174_TC** | **rs 6773957_AG** | **rs1063537_CT** | **rs2082940_TC** | **rs4686804_GA** | **rs1063539_GC** | **rs9842733_AT** |
| FT* | 1 | 40 | 2 | 6.1* | 0.8 | 4 | 1.06 | GT | TT | AA | CT | TC | GG | GC | AA |
| MT** | 2 | 40 | 1 | 6.3** | 0.5 | 4.2** | 1.04 | GT | TT | AG | CT | TC | GA | GC | AA |
| S1 | 1 | 20 | 2 | 5.3 | 0.4 | 3.6 | 0.86** | GT | TT | AA | CT | TC | GG | GC | AA |
| S2 | 1 | 17 | 0 | 3.1 | 0.2 | 1.5 | 1.35 | TT | TT | AG | CC | CC | GA | CC | AA |
| **S3***** | 1 | 14 | 1 | 10.1** | 0.3 | 7.9** | 0.51** | GG | TT | AG | TT | TT | GG | GG | AA |
| S4*** | 1 | 12 | 1 | 6.2* | 1.0 | 4.4** | 0.98** | GT | TT | AA | CT | TC | GG | GC | AA |
| S5 | 1 | 10 | 0 | 4.7 | 0.6 | 2.6 | 1.36 | GT | TT | AA | CT | TC | GG | GC | AA |
| S6*** | 1 | 9 | 1 | 6.9** | 1 | 4.6** | 1.05 | GT | TT | AG | CT | TC | GA | GC | AA |
| S7 | 1 | 3 | 0 | 3.3 | 0.5 | 1.4 | 1.61 | GG | TT | AA | TT | TT | GG | GG | AA |
| D1 | 2 | 5 | 1 | 6.2* | 0.7 | 4.3** | 1.00** | GT | TT | AA | TT | TT | GG | GG | AA |
| D2*** | 2 | 15 | 2 | 4.8 | 0.8 | 3.2 | 0.67** | GG | TT | AG | CC | CC | GA | CC | AA |
| The table shows the lipid profiles of the family, with the primary proband displaying very high total cholesterol (Chol) and low density lipoprotein-cholesterol (LDL-Chol) as well as low high density lipoprotein-cholesterol (HDL-Chol) levels. Affected members are marked in red. Lipid level ranges are: Total Cholesterol, total (Desirable: <5.2 mmol/L; Borderline high: 5.2-6.2 mmol/L; High: ≥6.2 mmol/L); Triglycerides (Normal: <1.7 mmol/L; Borderline high: 1.7-2.25 mmol/L; High: 2.26-5.64 mmol/L; Very high:≥5.65 mmol/L); LDL-cholesterol (Optimal: <2.59 mmol/L; Low risk: 2.59–3.34 mmol/L; Borderline high: 3.37–4.12 mmol/L; high: 4.14–4.90 mmol/L; Very high: ≥4.92 mmol/L) and HDL-Cholesterol (Low HDL: <1.04 mmol/L; Normal: 1.04–1.55 mmol/L; Desirable: >1.55 mmol/L); *Borderline high total cholesterol levels; **Out-of range levels. Genotype results are shown only for the variants studied in the general population. FT, father; MT, mother, S, son; D, daughter | | | | | | | | | | | | | | | |

| **Suppl. Table 2:** Association of CAD with cardiovascular disease traits and ADIPOQ variants | | | | | |
| --- | --- | --- | --- | --- | --- |
| **Model** | **Unstandardized Coefficients** | | **Standardized Coefficients** | **t** | **P-value** |
|  | B | S.E. | Beta |  |  |
| (Constant) | .022 | .096 |  | .229 | .819 |
| rs2241766G | .394 | .124 | .169 | 3.169 | .002 |
| rs6773957G | -.269 | .133 | -.266 | -2.028 | .043 |
| rs6444174C | .157 | .128 | .070 | 1.227 | .220 |
| rs1063537T | -.090 | .101 | -.069 | -.894 | .371 |
| rs2082940T | -.136 | .078 | -.112 | -1.752 | .080 |
| rs4686804T | .077 | .108 | .075 | .714 | .475 |
| rs1063539C | .207 | .119 | .162 | 1.741 | .082 |
| rs9842733T | -.345 | .124 | -.110 | -2.773 | .006 |
| rs2241766T | .395 | .123 | .176 | 3.214 | .001 |
| rs6773957A | -.289 | .132 | -.286 | -2.186 | .029 |
| rs6444174T | .184 | .126 | .083 | 1.462 | .144 |
| rs1063537C | -.083 | .099 | -.064 | -.841 | .400 |
| rs2082940C | -.137 | .076 | -.115 | -1.809 | .071 |
| rs4686804C | .096 | .108 | .093 | .885 | .376 |
| rs1063539G | .200 | .119 | .157 | 1.683 | .092 |
| rs9842733A | -.299 | .120 | -.099 | -2.500 | .012 |
| Sex | -.109 | .009 | -.104 | -12.392 | .000 |
| Age | .003 | .000 | .082 | 8.735 | .000 |
| DM | .084 | .009 | .084 | 9.429 | .000 |
| MI | .584 | .010 | .557 | 60.330 | .000 |
| HTN | .032 | .010 | .027 | 3.092 | .002 |
| OBS | -.020 | .008 | -.019 | -2.375 | .018 |
|  | | | | | |

| **Suppl. Table 3.** Association of myocardial infarction with ADIPOQ gene variants in relation to coronary artery disease, as a dependent variable | | | | | | |
| --- | --- | --- | --- | --- | --- | --- |
| Parameter | Beta | S.E. | t | P-value | 95% Confidence Interval | |
|  |  |  |  |  | Lower Bound | Upper Bound |
| Intercept | .755 | .107 | 7.052 | .000 | .545 | .965 |
| [MI=0] * rs2241766G | -.893 | .425 | -2.102 | .036 | -1.725 | -.060 |
| [MI=1] * rs2241766G | .453 | .161 | 2.820 | .005 | .138 | .769 |
| [MI=0] * rs2241766T | -.941 | .423 | -2.225 | .026 | -1.771 | -.112 |
| [MI=1] * rs2241766T | .483 | .159 | 3.029 | .002 | .170 | .795 |
| [MI=0] * rs6773957G | .956 | .576 | 1.658 | .097 | -.174 | 2.085 |
| [MI=1] * rs6773957G | -.101 | .162 | -.626 | .531 | -.418 | .215 |
| [MI=0] * rs6773957A | .941 | .576 | 1.634 | .102 | -.188 | 2.071 |
| [MI=1] * rs6773957A | -.117 | .161 | -.730 | .466 | -.433 | .198 |
| [MI=0] * rs6444174C | -.623 | .447 | -1.392 | .164 | -1.500 | .254 |
| [MI=1] * rs6444174C | .409 | .179 | 2.288 | .022 | .059 | .760 |
| [MI=0] * rs6444174T | -.614 | .445 | -1.381 | .167 | -1.487 | .258 |
| [MI=1] * rs6444174T | .436 | .176 | 2.476 | .013 | .091 | .781 |
| [MI=0] * rs1063537T | .330 | .357 | .924 | .355 | -.370 | 1.030 |
| [MI=1] * rs1063537T | -.320 | .147 | -2.172 | .030 | -.609 | -.031 |
| [MI=0] * rs1063537C | .359 | .355 | 1.010 | .313 | -.338 | 1.056 |
| [MI=1] * rs1063537C | -.287 | .146 | -1.969 | .049 | -.573 | -.001 |
| [MI=0] * rs2082940T | .217 | .183 | 1.182 | .237 | -.143 | .576 |
| [MI=1] * rs2082940T | -.046 | .097 | -.480 | .631 | -.236 | .143 |
| [MI=0] * rs2082940C | .208 | .178 | 1.163 | .245 | -.142 | .557 |
| [MI=1] * rs2082940C | -.049 | .095 | -.516 | .606 | -.234 | .137 |
| [MI=0] * rs4686804T | -.135 | .329 | -.409 | .682 | -.780 | .510 |
| [MI=1] * rs4686804T | -.045 | .151 | -.300 | .764 | -.341 | .250 |
| [MI=0] * rs4686804C | -.115 | .329 | -.350 | .727 | -.761 | .530 |
| [MI=1] * rs4686804C | -.028 | .151 | -.187 | .851 | -.325 | .268 |
| [MI=0] * rs1063539C | -.450 | .284 | -1.582 | .114 | -1.007 | .108 |
| [MI=1] * rs1063539C | .205 | .161 | 1.272 | .203 | -.111 | .521 |
| [MI=0] * rs1063539G | -.474 | .282 | -1.679 | .093 | -1.027 | .079 |
| [MI=1] * rs1063539G | .178 | .161 | 1.103 | .270 | -.138 | .494 |
| [MI=0] * rs9842733T | -.090 | .227 | -.395 | .693 | -.534 | .355 |
| [MI=1] * rs9842733T | -.638 | .186 | -3.422 | .001 | -1.003 | -.272 |
| [MI=0] * rs9842733A | -.025 | .219 | -.116 | .908 | -.455 | .404 |
| [MI=1] * rs9842733A | -.620 | .181 | -3.419 | .001 | -.975 | -.265 |
| The table shows interactive relationships for the adiponectin Q SNPs with myocardial infarction on coronary artery disease determined by ANOVA. In the parameter column, 0 denotes controls and 1 denotes cases. Beta represents the statistical power of the test. MI, myocardial infarction; S.E., standard error; t, t-test. | | | | | | |

| **Suppl. Table 4.** Association of hypertension with gene variants in relation to coronary artery disease as the dependent variable. | | | | | | |
| --- | --- | --- | --- | --- | --- | --- |
| Parameter | Beta | S.E. | t | P-value | 95% Confidence Interval | |
|  |  |  |  |  | Lower Bound | Upper Bound |
| Intercept | .754 | .130 | 5.791 | .000 | .499 | 1.009 |
| [HTN=0] * rs2241766G | .805 | .385 | 2.089 | .037 | .050 | 1.560 |
| [HTN=1] * rs2241766G | -.020 | .212 | -.096 | .923 | -.436 | .395 |
| [HTN=0] * rs2241766T | .805 | .382 | 2.107 | .035 | .056 | 1.555 |
| [HTN=1] * rs2241766T | -.035 | .210 | -.168 | .866 | -.447 | .376 |
| [HTN=0] * rs6773957G | -.161 | .389 | -.414 | .679 | -.924 | .602 |
| [HTN=1] * rs6773957G | .058 | .216 | .271 | .787 | -.365 | .481 |
| [HTN=0] * rs6773957A | -.179 | .387 | -.463 | .643 | -.937 | .579 |
| [HTN=1] * rs6773957A | .048 | .215 | .221 | .825 | -.375 | .470 |
| [HTN=0] * rs6444174C | -.653 | .435 | -1.500 | .134 | -1.507 | .200 |
| [HTN=1] * rs6444174C | -.167 | .190 | -.880 | .379 | -.540 | .205 |
| [HTN=0] * rs6444174T | -.573 | .429 | -1.334 | .182 | -1.414 | .269 |
| [HTN=1] * rs6444174T | -.157 | .186 | -.842 | .400 | -.522 | .208 |
| [HTN=0] * rs1063537T | -.387 | .293 | -1.324 | .185 | -.961 | .186 |
| [HTN=1] * rs1063537T | .121 | .163 | .741 | .459 | -.199 | .441 |
| [HTN=0] * rs1063537C | -.376 | .287 | -1.309 | .190 | -.938 | .187 |
| [HTN=1] * rs1063537C | .131 | .161 | .818 | .413 | -.184 | .447 |
| [HTN=0] * rs2082940T | -.073 | .151 | -.482 | .630 | -.369 | .223 |
| [HTN=1] * rs2082940T | -.292 | .141 | -2.066 | .039 | -.569 | -.015 |
| [HTN=0] * rs2082940C | -.140 | .139 | -1.009 | .313 | -.413 | .132 |
| [HTN=1] * rs2082940C | -.278 | .140 | -1.983 | .047 | -.553 | -.003 |
| [HTN=0] * rs4686804T | .583 | .289 | 2.020 | .043 | .017 | 1.148 |
| [HTN=1] * rs4686804T | .201 | .178 | 1.126 | .260 | -.149 | .550 |
| [HTN=0] * rs4686804C | .624 | .287 | 2.177 | .030 | .062 | 1.187 |
| [HTN=1] * rs4686804C | .212 | .179 | 1.184 | .237 | -.139 | .564 |
| [HTN=0] * rs1063539C | -.754 | .370 | -2.039 | .042 | -1.479 | -.029 |
| [HTN=1] * rs1063539C | .340 | .192 | 1.772 | .076 | -.036 | .717 |
| [HTN=0] * rs1063539G | -.769 | .375 | -2.053 | .040 | -1.504 | -.035 |
| [HTN=1] * rs1063539G | .314 | .191 | 1.646 | .100 | -.060 | .688 |
| [HTN=0] * rs9842733T | .171 | .394 | .433 | .665 | -.602 | .943 |
| [HTN=1] * rs9842733T | -.436 | .183 | -2.381 | .017 | -.795 | -.077 |
| [HTN=0] * rs9842733A | .195 | .382 | .509 | .611 | -.555 | .944 |
| [HTN=1] * rs9842733A | -.411 | .177 | -2.325 | .020 | -.757 | -.064 |
| The table shows interactive relationships for the adiponectin Q SNPs with hypertension on coronary artery disease. In the parameter column, 0 denotes controls and 1 denotes cases. Beta represents the statistical power of the test. S.E., standard error; t, t-test. | | | | | | |

| **Suppl. Table 5.** Association of type 2 diabetes mellitus with gene variants in relations to coronary artery disease as a dependent variable | | | | | | |
| --- | --- | --- | --- | --- | --- | --- |
| **Parameter** | Beta | S. E. | t | P-value | **95% Confidence Interval** | |
|  |  |  |  |  | **Lower Bound** | **Upper Bound** |
| Intercept | .703 | .133 | 5.274 | .000 | .442 | .964 |
| [DM=0] * rs2241766G | .281 | .257 | 1.090 | .276 | -.224 | .785 |
| [DM=1] * rs2241766G | -.068 | .244 | -.277 | .782 | -.547 | .411 |
| [DM=0] * rs2241766T | .281 | .255 | 1.105 | .269 | -.218 | .780 |
| [DM=1] * rs2241766T | -.067 | .242 | -.278 | .781 | -.542 | .407 |
| [DM=0] * rs6773957G | -.356 | .279 | -1.275 | .202 | -.902 | .191 |
| [DM=1] * rs6773957G | -.406 | .286 | -1.423 | .155 | -.966 | .153 |
| [DM=0] * rs6773957A | -.393 | .279 | -1.409 | .159 | -.939 | .154 |
| [DM=1] * rs6773957A | -.405 | .285 | -1.424 | .155 | -.963 | .153 |
| [DM=0] * rs6444174C | .151 | .282 | .536 | .592 | -.401 | .703 |
| [DM=1] * rs6444174C | .086 | .261 | .330 | .742 | -.426 | .598 |
| [DM=0] * rs6444174T | .193 | .277 | .697 | .486 | -.350 | .736 |
| [DM=1] * rs6444174T | .106 | .258 | .410 | .682 | -.399 | .611 |
| [DM=0] * rs1063537T | -.025 | .224 | -.110 | .912 | -.464 | .415 |
| [DM=1] * rs1063537T | .135 | .167 | .808 | .419 | -.192 | .462 |
| [DM=0] * rs1063537C | -.080 | .220 | -.364 | .716 | -.512 | .352 |
| [DM=1] * rs1063537C | .195 | .165 | 1.180 | .238 | -.129 | .519 |
| [DM=0] * rs2082940T | -.240 | .121 | -1.980 | .048 | -.478 | -.002 |
| [DM=1] * rs2082940T | -.357 | .227 | -1.576 | .115 | -.801 | .087 |
| [DM=0] * rs2082940C | -.217 | .115 | -1.891 | .059 | -.441 | .008 |
| [DM=1] * rs2082940C | -.387 | .226 | -1.714 | .087 | -.831 | .056 |
| [DM=0] * rs4686804T | .317 | .190 | 1.671 | .095 | -.055 | .689 |
| [DM=1] * rs4686804T | .175 | .230 | .758 | .448 | -.277 | .626 |
| [DM=0] * rs4686804C | .347 | .191 | 1.814 | .070 | -.028 | .723 |
| [DM=1] * rs4686804C | .191 | .230 | .831 | .406 | -.259 | .641 |
| [DM=0] * rs1063539C | .327 | .297 | 1.102 | .270 | -.255 | .909 |
| [DM=1] * rs1063539C | .210 | .222 | .945 | .345 | -.226 | .646 |
| [DM=0] * rs1063539G | .349 | .294 | 1.190 | .234 | -.226 | .925 |
| [DM=1] * rs1063539G | .165 | .223 | .739 | .460 | -.272 | .602 |
| [DM=0] * rs9842733T | -.796 | .304 | -2.618 | .009 | -1.392 | -.200 |
| [DM=1] * rs9842733T | .090 | .222 | .404 | .686 | -.345 | .525 |
| [DM=0] * rs9842733A | -.823 | .298 | -2.764 | .006 | -1.406 | -.239 |
| [DM=1] * rs9842733A | .155 | .215 | .723 | .470 | -.266 | .577 |
| The table shows interactive relationships for the adiponectin Q SNPs with type 2 diabetes mellitus on coronary artery disease. In the parameter column, 0 denotes controls and 1 denotes cases. Beta represents the statistical power of the test. S.E., standard error; t, standard t-test. | | | | | | |

| **Suppl. Table 6.** Association of gender with gene variants in relations to coronary artery disease as the dependent variable | | | | | |
| --- | --- | --- | --- | --- | --- |
| **Source** | **Type III Sum of Squares** | **df** | **Mean Square** | **F** | **Sig.** |
| Corrected Model | 144.353^a^ | 32 | 4.511 | 19.211 | .000 |
| Intercept | 5.066 | 1 | 5.066 | 21.576 | .000 |
| Sex * rs2241766G | .365 | 2 | .183 | .778 | .460 |
| Sex * rs2241766T | .306 | 2 | .153 | .652 | .521 |
| Sex * rs6773957G | 1.469 | 2 | .734 | 3.127 | .044 |
| Sex * rs6773957A | 1.418 | 2 | .709 | 3.020 | .049 |
| Sex * rs6444174C | .389 | 2 | .194 | .828 | .437 |
| Sex * rs6444174T | .456 | 2 | .228 | .970 | .379 |
| Sex * rs1063537T | .518 | 2 | .259 | 1.103 | .332 |
| Sex * rs1063537C | .354 | 2 | .177 | .754 | .470 |
| Sex * rs2082940T | 1.856 | 2 | .928 | 3.951 | .019 |
| Sex * rs2082940C | 1.875 | 2 | .938 | 3.993 | .018 |
| Sex * rs4686804T | .376 | 2 | .188 | .801 | .449 |
| Sex * rs4686804C | .516 | 2 | .258 | 1.099 | .333 |
| Sex * rs1063539C | .331 | 2 | .166 | .706 | .494 |
| Sex * rs1063539G | .210 | 2 | .105 | .447 | .640 |
| Sex * rs9842733T | .173 | 2 | .086 | .368 | .692 |
| Sex * rs9842733A | .196 | 2 | .098 | .417 | .659 |
| a. R Squared = .062 (Adjusted R Squared = .059); | | | | | |

| **Suppl. Table 7.** Association of age with ADIPOQ gene variants in relation to coronary artery disease, as a dependent variable | | | | | | |
| --- | --- | --- | --- | --- | --- | --- |
| **Parameter** | **Beta** | **S. E.** | **t** | **Sig.** | **95% Confidence Interval** | |
|  |  |  |  |  | **Lower Bound** | **Upper Bound** |
| Intercept | .703 | .135 | 5.222 | .000 | .439 | .967 |
| [AgeUp=1] * rs2241766G | .196 | .266 | .740 | .460 | -.324 | .717 |
| [AgeUp=2] * rs2241766G | .069 | .201 | .343 | .732 | -.325 | .462 |
| [AgeUp=1] * rs2241766T | .260 | .260 | 1.000 | .318 | -.250 | .771 |
| [AgeUp=2] * rs2241766T | .043 | .199 | .218 | .828 | -.346 | .433 |
| [AgeUp=1] * rs6773957G | .023 | .042 | .549 | .583 | -.060 | .106 |
| [AgeUp=2] * rs6773957G | -.332 | .194 | -1.708 | .088 | -.712 | .049 |
| [AgeUp=1] * rs6773957A | 0^a^ | . | . | . | . | . |
| [AgeUp=2] * rs6773957A | -.353 | .194 | -1.820 | .069 | -.732 | .027 |
| [AgeUp=1] * rs6444174C | -.287 | .327 | -.876 | .381 | -.928 | .355 |
| [AgeUp=2] * rs6444174C | .161 | .239 | .675 | .500 | -.307 | .629 |
| [AgeUp=1] * rs6444174T | -.293 | .319 | -.920 | .357 | -.918 | .332 |
| [AgeUp=2] * rs6444174T | .195 | .237 | .822 | .411 | -.269 | .658 |
| [AgeUp=1] * rs1063537T | .230 | .214 | 1.073 | .283 | -.190 | .649 |
| [AgeUp=2] * rs1063537T | .055 | .195 | .285 | .776 | -.327 | .437 |
| [AgeUp=1] * rs1063537C | .187 | .204 | .918 | .358 | -.212 | .586 |
| [AgeUp=2] * rs1063537C | .095 | .195 | .484 | .628 | -.288 | .477 |
| [AgeUp=1] * rs2082940T | -.283 | .158 | -1.786 | .074 | -.593 | .028 |
| [AgeUp=2] * rs2082940T | -.152 | .130 | -1.173 | .241 | -.407 | .102 |
| [AgeUp=1] * rs2082940C | -.219 | .146 | -1.503 | .133 | -.505 | .067 |
| [AgeUp=2] * rs2082940C | -.176 | .128 | -1.376 | .169 | -.428 | .075 |
| [AgeUp=1] * rs4686804T | -.008 | .205 | -.037 | .970 | -.409 | .394 |
| [AgeUp=2] * rs4686804T | .011 | .259 | .042 | .966 | -.497 | .519 |
| [AgeUp=1] * rs4686804C | .025 | .205 | .122 | .903 | -.377 | .427 |
| [AgeUp=2] * rs4686804C | .029 | .260 | .110 | .912 | -.480 | .537 |
| [AgeUp=1] * rs1063539C | .043 | .234 | .185 | .853 | -.415 | .502 |
| [AgeUp=2] * rs1063539C | .309 | .234 | 1.319 | .187 | -.150 | .768 |
| [AgeUp=1] * rs1063539G | -.054 | .236 | -.228 | .820 | -.516 | .409 |
| [AgeUp=2] * rs1063539G | .299 | .234 | 1.281 | .200 | -.159 | .757 |
| [AgeUp=1] * rs9842733T | -.344 | .281 | -1.227 | .220 | -.894 | .206 |
| [AgeUp=2] * rs9842733T | -.251 | .223 | -1.123 | .261 | -.688 | .187 |
| [AgeUp=1] * rs9842733A | -.382 | .268 | -1.426 | .154 | -.908 | .143 |
| [AgeUp=2] * rs9842733A | -.228 | .218 | -1.049 | .294 | -.655 | .199 |
| ^a^This parameter is set to zero because it is redundant. Beta represents the statistical power of the test. S. E., standard error; t, standard t-test. AgeUp1<=45; AgeUP2>45 | | | | | | |

| **Suppl. Table 8.** Effect of male gender and age (cut-off >=45) on the relationship of CAD with disease traits and gene variants | | | | | | | |
| --- | --- | --- | --- | --- | --- | --- | --- |
| **Model** | **Unstandardized Coefficients** | | **Standardized Coefficients** | **t** | **P-value** | **95.0% Confidence Interval for Beta** | |
|  | **Beta** | **S.E.** | **Beta** |  |  | **Lower Bound** | **Upper Bound** |
| (Constant) | .342 | .194 |  | 1.765 | .078 | -.038 | .723 |
| DM | .076 | .012 | .080 | 6.169 | .000 | .052 | .100 |
| MI | .598 | .015 | .506 | 39.781 | .000 | .569 | .627 |
| HTN | .043 | .015 | .036 | 2.810 | .005 | .013 | .074 |
| OBS | -.009 | .012 | -.009 | -.723 | .470 | -.033 | .015 |
| rs2241766G | .411 | .209 | .191 | 1.970 | .049 | .002 | .821 |
| rs6773957G | -.223 | .226 | -.240 | -.991 | .322 | -.666 | .219 |
| rs6444174C | .473 | .364 | .231 | 1.300 | .194 | -.240 | 1.187 |
| rs1063537T | -.324 | .200 | -.273 | -1.620 | .105 | -.715 | .068 |
| rs2082940T | -.269 | .186 | -.245 | -1.450 | .147 | -.633 | .095 |
| rs4686804T | .111 | .331 | .117 | .336 | .737 | -.538 | .760 |
| rs1063539C | .399 | .361 | .342 | 1.106 | .269 | -.309 | 1.107 |
| rs9842733T | -.880 | .406 | -.316 | -2.165 | .030 | -1.676 | -.083 |
| rs2241766T | .389 | .207 | .186 | 1.874 | .061 | -.018 | .795 |
| rs6773957A | -.247 | .224 | -.266 | -1.102 | .270 | -.687 | .193 |
| rs6444174T | .522 | .363 | .261 | 1.437 | .151 | -.190 | 1.233 |
| rs1063537C | -.292 | .200 | -.248 | -1.457 | .145 | -.685 | .101 |
| rs2082940C | -.255 | .183 | -.233 | -1.392 | .164 | -.613 | .104 |
| rs4686804C | .132 | .332 | .139 | .397 | .692 | -.518 | .781 |
| rs1063539G | .358 | .359 | .307 | .996 | .319 | -.346 | 1.063 |
| rs9842733A | -.812 | .403 | -.296 | -2.016 | .044 | -1.603 | -.022 |
| a. Dependent Variable: CAD; Beta represents the statistical power of the test. S.E., standard error; t, standard t-test | | | | | | | |

| **Suppl. Table 9.** Effect of female gender and age (cut-off >=50) on the relationship of CAD with disease traits and gene variants | | | | | | | |
| --- | --- | --- | --- | --- | --- | --- | --- |
| **Model** | **Unstandardized Coefficients** | | **Standardized Coefficients** | **t** | **P-value** | **95.0% Confidence Interval for Beta** | |
|  | **Beta** | **S.E.** | **Beta** |  |  | **Lower Bound** | **Upper Bound** |
| (Constant) | .307 | .290 |  | 1.056 | .291 | -.263 | .876 |
| DM | .099 | .021 | .093 | 4.682 | .000 | .058 | .141 |
| MI | .580 | .020 | .553 | 28.570 | .000 | .540 | .620 |
| HTN | .014 | .029 | .009 | .481 | .630 | -.044 | .072 |
| OBS | -.051 | .019 | -.051 | -2.658 | .008 | -.089 | -.013 |
| rs2241766G | -.006 | .504 | -.003 | -.013 | .990 | -.995 | .982 |
| rs6444174C | -.057 | .446 | -.024 | -.128 | .898 | -.932 | .818 |
| rs1063537T | .155 | .292 | .119 | .533 | .594 | -.417 | .727 |
| rs2082940T | -.010 | .172 | -.008 | -.057 | .955 | -.348 | .328 |
| rs4686804T | -.047 | .042 | -.046 | -1.128 | .260 | -.128 | .035 |
| rs1063539C | .015 | .442 | .012 | .034 | .973 | -.852 | .882 |
| rs9842733T | -.345 | .533 | -.107 | -.648 | .517 | -1.391 | .700 |
| rs2241766T | .018 | .502 | .008 | .037 | .971 | -.965 | 1.002 |
| rs6773957A | -.059 | .041 | -.059 | -1.449 | .148 | -.139 | .021 |
| rs6444174T | -.071 | .443 | -.031 | -.161 | .872 | -.941 | .798 |
| rs1063537C | .132 | .291 | .103 | .454 | .650 | -.439 | .703 |
| rs2082940C | -.053 | .171 | -.044 | -.308 | .758 | -.387 | .282 |
| rs1063539G | .091 | .442 | .073 | .206 | .837 | -.776 | .958 |
| rs9842733A | -.308 | .530 | -.099 | -.582 | .561 | -1.347 | .731 |
| a. Dependent Variable: CAD; Beta represents the statistical power of the test. S.E., standard error; t, standard t-test | | | | | | | |
